# Regression of cardiac amyloid load documented by cardiovascular magnetic resonance in a patient with hereditary amyloidosis

**DOI:** 10.1007/s00392-020-01611-2

**Published:** 2020-02-11

**Authors:** Anca Florian, Michael Bietenbeck, Grigorios Chatzantonis, Anna Hüsing-Kabar, Hartmut Schmidt, Ali Yilmaz

**Affiliations:** 1 Department of Cardiology I, Division of Cardiovascular Imaging, University Hospital Münster, Albert-Schweitzer-Campus 1, Building A1, 48149 Münster, Germany; 2 Department of Gastroenterology/Hepatology, University Hospital Münster, Münster, Germany

Sirs:

We report the case of a 58-year old female with hereditary transthyretin amyloidosis (hATTR) due to a heterozygote mutation in the transthyretin (TTR) gene (*Gly47Ala*). An uncle, an aunt, and four first-degree cousins were also suffering from the same disease. At the present time-point (June 2019), she presented to our outpatient clinic (dedicated to patients with cardiomyopathies) for follow-up cardiac evaluation due to recurrent paroxysmal atrial fibrillation.

The genetic diagnosis had been confirmed at the age of 43 years and characteristic amyloidotic deposits were documented in a colon biopsy at that time. The patient had been suffering from a slowly progressive polyneuropathy for the past 8 years, for which she received therapy with tafamidis for almost 5 years in total (2012–2015 and 2016–2018—until transplantation), interrupted by inotersen (verum) within a double-blind randomized trial of inotersen vs. placebo for 65 weeks (2015–2016). Finally, in early 2018, the patient underwent successful second liver transplantation (LTx)—1 month after the first LTx graft failed due to portal vein thrombosis. A timeline of disease-modifying therapies and non-invasive imaging parameters is shown in Fig. 1. In addition, the patient suffered from chronic kidney disease and arterial hypertension. Over the years, she underwent repeated surgery for bilateral carpal tunnel syndrome.

**Fig. 1:**
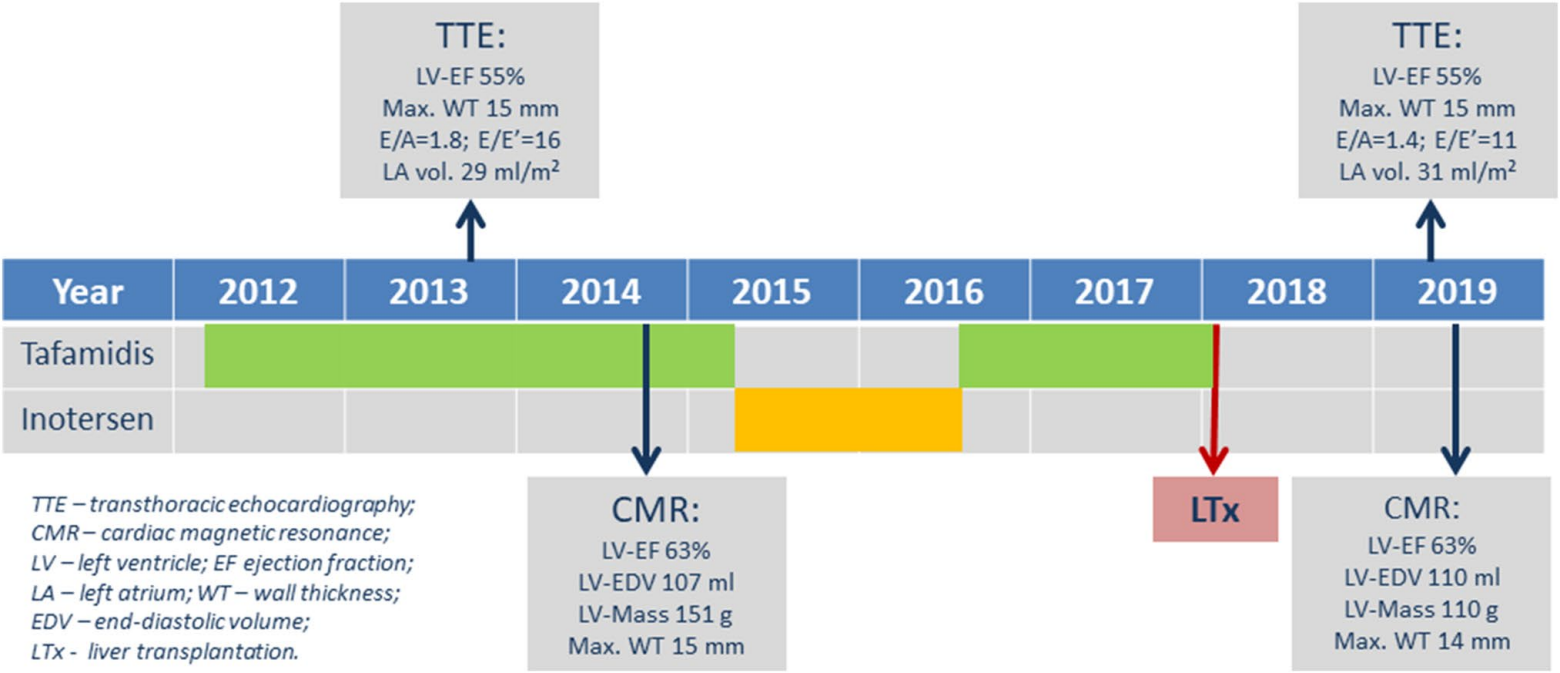
Diagram depicting the timeline of undergone disease-modifying therapies together with several relevant non-invasive imaging parameters measured at two time-points: beginning and present evaluation

Regarding her cardiac history, the patient started complaining of a slowly progressive reduction in exercise capacity approximately 7 years prior to current presentation. Repeated transthoracic echocardiography (TTE) (2013 and 2014; at that time on tafamidis therapy since 2012) had shown concentric left ventricular (LV) hypertrophy (max. septal wall thickness = 15 mm) (Fig. 2a, b) and diastolic dysfunction with signs of increased filling pressures. Cardiac involvement was confirmed by cardiovascular magnetic resonance imaging (CMR) already in 2014 (still on tafamidis) by depiction of extensive, circumferential (non-ischemic pattern) late gadolinium enhancement (LGE) in both ventricles a well as in the walls of both atria—a characteristic finding for cardiac amyloidosis (Fig. 3a, b). The LV was not dilated, but hypertrophied (max. 14–15 mm) (Fig. 4a, b) and showed normal systolic function (LV-EF 63%).

**Fig. 2:**
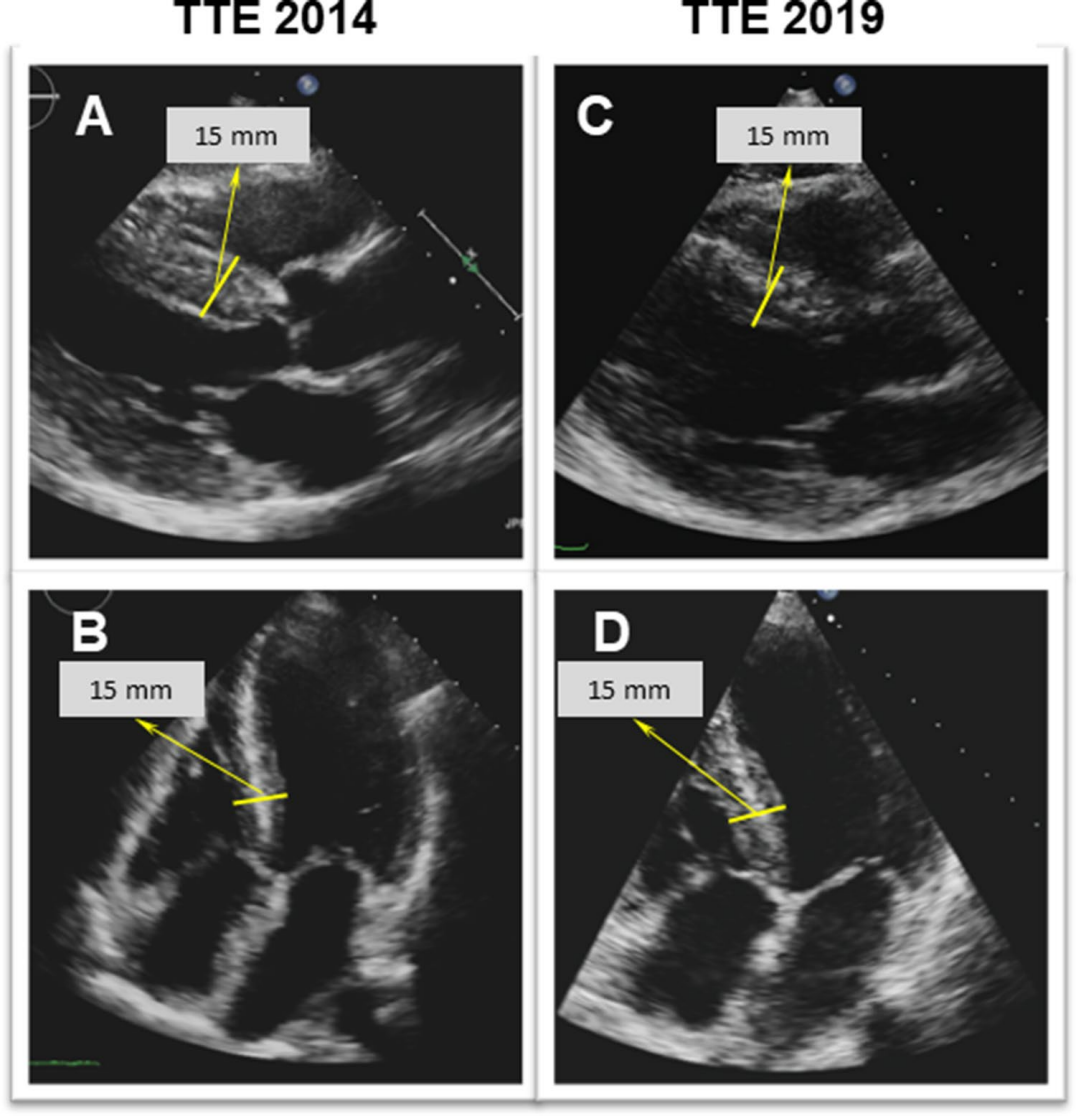
Two-dimensional transthoracic echocardiography still frames at end-diastole in parasternal long-axis (**a, c**) and apical four-chamber (**b**, **d**) views from year 2014 (around the first CMR examination; **a**, **b**) and at present evaluation (2019; **c**, **d**). Concentric LV hypertrophy can be seen at both time points—without any relevant progression from 2014 to 2019

**Fig. 3:**
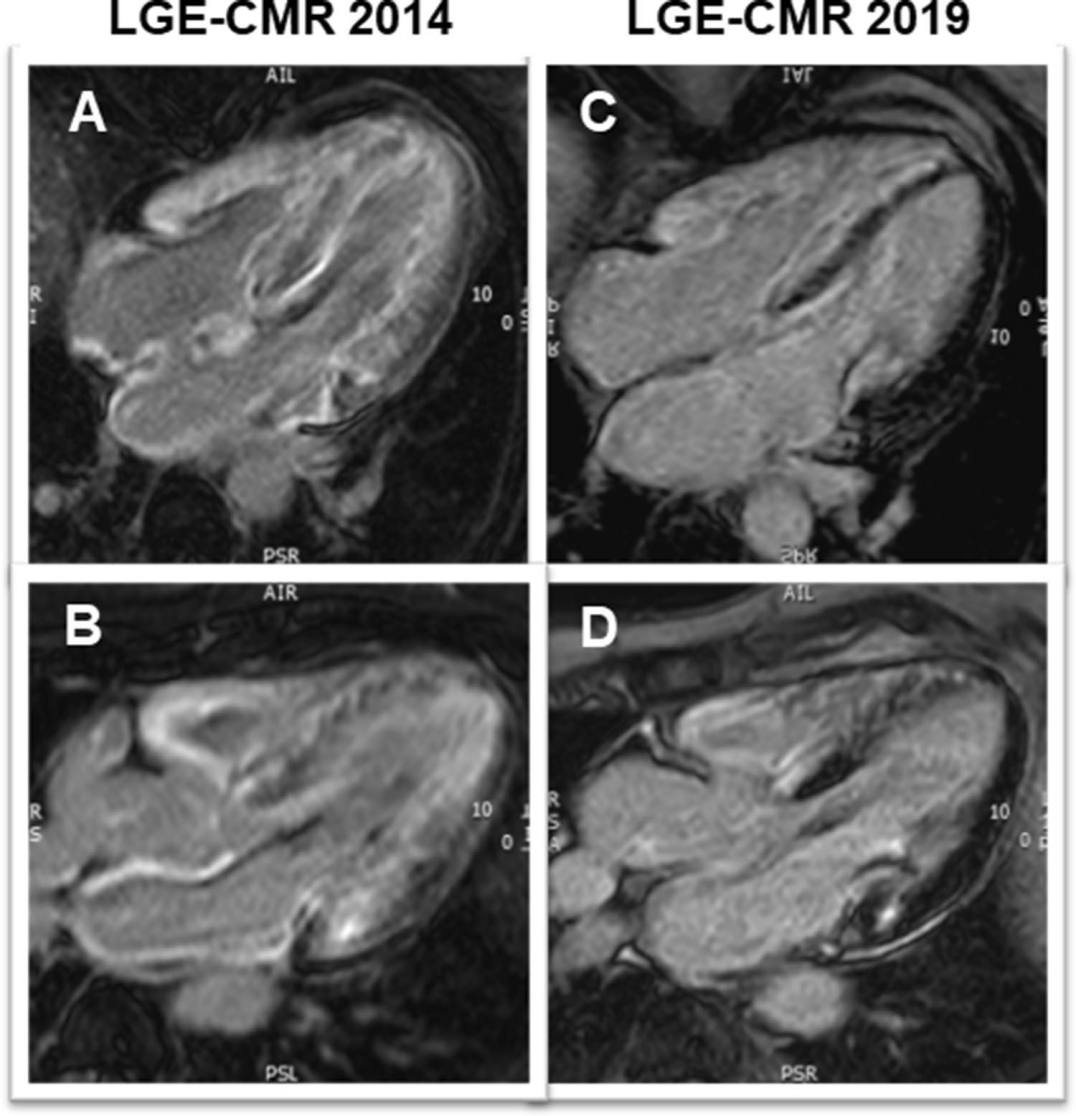
Late gadolinium enhancement (LGE) CMR images in four- (**a**, **c**) and three-chamber (**b**, **d**) views from first CMR (2014; **a**, **b**) and at present evaluation (2019; **c**, **d**). At first CMR (**a**, **b**) extensive, circumferential (non-ischemic pattern) LGE in both ventricles a well as in the walls of both atria—a characteristic finding for cardiac amyloidosis—can be seen. At present evaluation (**c**, **d**), an obvious decrease in the LGE extent, particularly in the lateral wall of the LV, in the subendocardium of the septum and in the atrial walls is noted

**Fig. 4:**
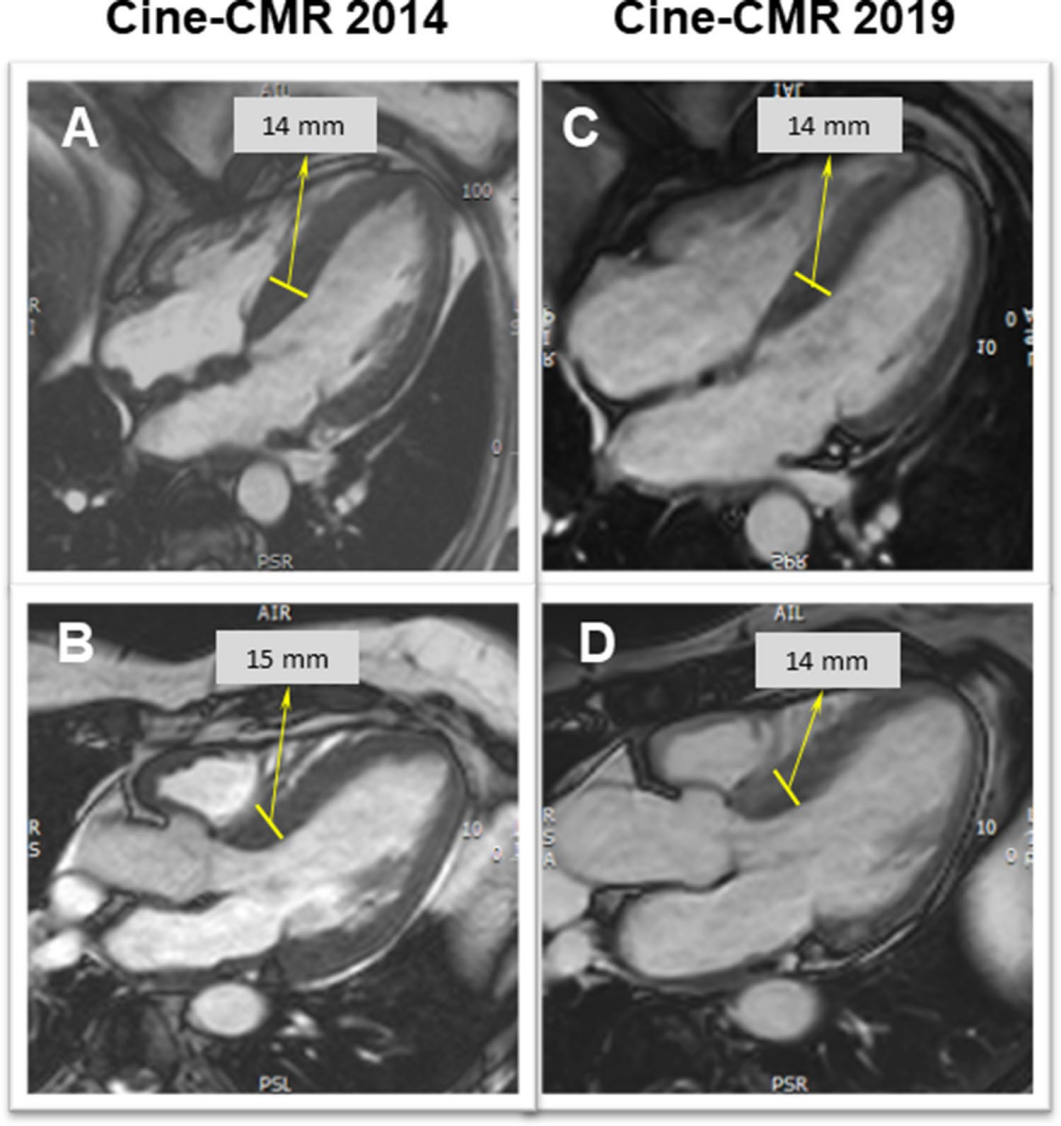
Diastolic cine-CMR still frames in four- (**a**, **c**) and three-chamber (**b**, **d**) views from first CMR (2014; **a**, **b**) and at present evaluation (2019; **c**, **d**). LV hypertrophy can be seen at both time points, without relevant progression. Noteworthy, the thickening of the atrial septum seen at first CMR (**a**) had disappeared at follow-up (**c**)

At the current cardiac examination, 16 months after the second LTx, the patient was in good general condition without overt cardiac symptoms (in particular no syncope and no edema)—in the context of limited mobility due to advanced polyneuropathy of lower and upper limbs and loss of sensibility in hands and feet. Walking was only possible using aids. Besides immunosuppressive therapy since her LTx in early 2018, the patient was currently taking a beta-blocker (2.5 mg bisoprolol/bid) due to previous detection of non-sustained ventricular tachycardia episodes (asymptomatic) in an earlier Holter ECG, a diuretic (torsemide, 10 mg qd) and a new oral anticoagulant (edoxaban, 30 mg qd). The patient received no ATTR disease-modifying therapy after LTx.

Clinical examination of the cardiovascular system was rather unremarkable. No signs of systemic or lung congestion were present. 12-lead resting ECG showed sinus rhythm (60 bpm), known trifascicular block: first degree AV-block (PR interval 260 ms), left bundle branch block (QRS duration 150 ms) with left axis deviation (QRS axis—60°); QTc 480 ms (Fig. 5). No overt low QRS voltage in the limb leads (< 5 mm) was present. Blood analysis revealed a stable elevated creatinine of 1.3 mg/dl in the context of chronic kidney disease, elevated natriuretic peptide (NT-proBNP of 1.849 pg/ml), and a mildly elevated high sensitive troponin T (80 ng/l).

**Fig. 5.**
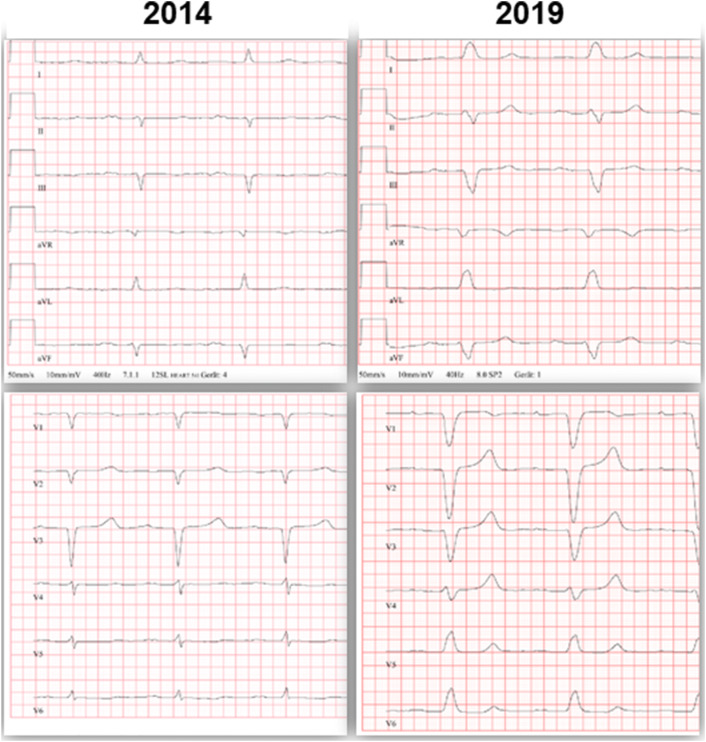
12-lead ECG tracings from year 2014 (around the first CMR examination; left hand panel) and at present evaluation (2019; right hand panel). In 2014. sinus rhythm (67 bpm), first degree AV-block (PR interval 240 ms), left anterior fascicular block (QRS duration 90 ms) with left axis deviation (QRS axis –55°) were present. At current evaluation a trifascicular block had developed: slight progression of first degree AV-block (PR interval 260 ms), now with complete left bundle branch block (QRS duration 150 ms)

Repeated TTE revealed a LV with rather unchanged concentric hypertrophy (max. septal wall thickness = 15 mm), preserved LV systolic function (LV-EF = 55%) (Fig. 2c, d) and grade I diastolic dysfunction (*E*/*A* = 1.4; average *E*/*E*' = 11; LA volume index 31 ml/m^2^). To better characterize the severity of cardiomyopathy, a follow-up CMR study was performed. The CMR results revealed the aforementioned septal LV hypertrophy (max. 14 mm) as well as mild RV hypertrophy (5 mm) and preserved biventricular systolic function (LV-EF 63%; RV-EF 62%) (Fig. 4c, d). Noteworthy, in direct comparison to the previous CMR study performed 5 years ago, there was a slight decrease in absolute (151–110 g) and indexed (78–63 g/m^2^) LV mass with constant LV end-diastolic volume (55–63 ml/m^2^) (Fig. 4). Feature-tracking based global as well as basal peak longitudinal strain remained impaired when compared to 2014 (–13% to –15%), but showed a subtle improvement regarding midventricular and apical segments (Fig. 6). Most surprisingly, in the post-contrast sequences, there was an impressive decrease in the extent of LGE, particularly in the lateral wall of the LV, in the subendocardium of the septum and in the atrial walls. Interestingly, the previous thickening of the atrial septum had almost completely disappeared (Figs. 3, 4).

**Fig. 6:**
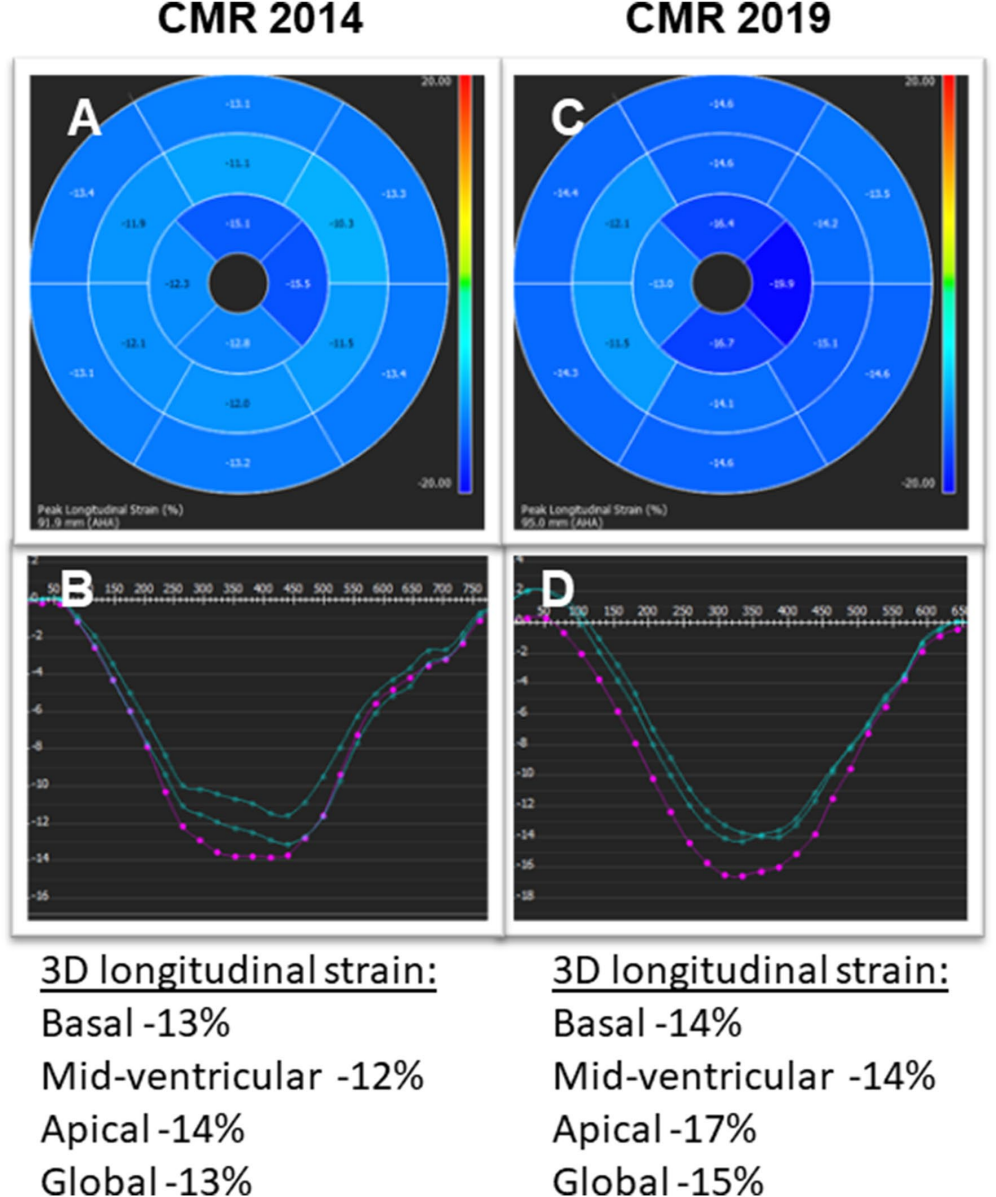
Three-dimensional (3D) LV longitudinal systolic strain values, measured by feature-tracking technique in the cine-CMR images, displayed as 16-segment (AHA model) bulls-eye (**a**, **c**) with the corresponding curves averaged at basal/mid-ventricular and apical LV cavity level—at first CMR (year 2014; **a**, **b**) and at present evaluation (2019; **c**, **d**). Reduced, rather unchanged global and regional longitudinal strain values are measured at both time points

These CMR-based findings suggested an unexpected and quite impressive improvement regarding the extent of cardiac amyloid deposits. Antiarrhythmic therapy with a beta-blocker was further indicated, together with the other previous medication, including oral anticoagulation. In addition, periodic cardiological follow-up including monitoring by Holter ECG for atrial/ventricular tachyarrhythmia or advanced conduction blocks was recommended.

Hereditary ATTR amyloidosis is an autosomal dominant, systemic disease that usually presents with progressive neuropathy and/or cardiomyopathy and is caused by mutations in the gene that encode transthyretin [1]. Over 130 pathogenic mutations in this gene have been identified and hence, the disease is characterized by a large phenotypic heterogeneity regarding onset and progression [1]. Diagnosis is based on TTR gene sequencing to detect causal mutation and biopsy to detect amyloid deposits [1]. So far, approved disease-modifying (anti-amyloid) therapies include (1) liver transplantation (LTx), (2) TTR stabilizers, including tafamidis and diflunisal and (3) TTR gene-silencing, including the antisense nucleotide inotersen and the small-interfering RNA, patisiran [1−3]. Nevertheless, tafamidis, diflunisal as well as inotersen and patisiran are currently not approved for the therapy of hATTR-associated cardiomyopathy in the absence of polyneuropathy, up to the present moment in Europe.

Hereditary ATTR-associated cardiomyopathy usually manifests with a hypertrophic/restrictive phenotype, conduction disease and arrhythmias leading to heart failure, reduced quality of life and finally to death [4]. A non-invasive diagnosis of cardiac amyloidosis is possible either by multi-parametric CMR or repurposed bone scintigraphy [3, 5].

The striking particularity of our case report is the concurrent demonstration of (1) a clinically silent cardiomyopathy over the last 5–6 years (since first diagnosis of cardiac involvement in 2013), without heart failure hospitalizations or other cardiac events and (2) the noticed regression (!) in cardiac phenotype severity as assessed by CMR within the last 5 years. In principle, hATTR cardiomyopathy may indeed manifest with a slow clinical progression—but almost never shows a substantial regression of cardiac imaging findings as was documented in this case [3].

The *Gly47Ala* (p.Gly67Ala) mutation is rare, being described in 1% of patients with hATTR cardiac amyloidosis in Europe. In comparison, *Val30Met* (p.Val50Met) is by far the most frequent one, with a reported prevalence in Europe of 74% [6]. Data on the natural history of cardiac amyloidosis associated with the *Gly47Ala* mutation are scarce. This variant was identified in a larger pedigree in Northwest Germany revealing a combined presentation of neurological and cardiac phenotype. Major cause of death is sudden death between 40 and 50 years of age among affected family members. A few studies including small numbers of patients with a *Gly47Ala* mutation showed the clinical predominance of neuropathy [7, 8]. In the THAOS European registry, patients with a mixed cardiac and neurologic involvement had a milder phenotype on echocardiography when compared to those with an isolated cardiac phenotype. Moreover, subjects with a non-cardiac mutation (which included *Gly47Ala)* had better survival than patients with cardiac mutations (*Val122Ile* (p.Val142Ile)*, Leu111Met* (p.Leu131Met)*, Thr60Ala* (p.Thr80Ala) *and Ile68Leu* (p.Ile88Leu) or wild-type (wt) ATTR amyloidosis, but worse when compared to *Val30Met* patients [6]. Notably, from the 186 patients in the non-cardiac mutation group at baseline, only two were alive at 6 years [6]. In another longitudinal study, patients with a non-*Val122Ile* amyloid cardiomyopathy had the highest median survival (69 months) when compared to *Val122Ile* or wtATTR cardiomyopathy (31 and 57 months, respectively)[4].

Importantly, our patient received a series of disease-modifying treatments. Sixteen months prior to the present evaluation, the patient underwent LTx which acts by suppressing the main source of mutant TTR [1]. Moreover, one may hypothesize that a reversal in transthyretin flux (transfer/evacuation of unbound transthyretin from the myocardium to the intravasal volume) may occur after successful LTx, since the concentration of transthyretin in the intravasal volume is tremendously decreased after LTx. However, with the current available scientific information, our hypothesis is based mainly on speculation. Nevertheless, according to two large registries, cardiac events were the leading cause of death after LTx in the long-term [9, 10]. In another study, an increase in LV septal thickness was seen on echocardiography 16 months after LTx even in patients with the *Val30Met* mutation [11]. Nevertheless, large differences in survival were observed in relation to different mutations and even between mutations with similar phenotypes [12].

Prior to LTx, the patient had received a tafamidis therapy for almost 5 years. Tafamidis, a TTR stabilizer, was shown to slow the progression of ATTR polyneuropathy and was approved for its treatment in numerous countries [3]. Moreover, in the ATTR-ACT trial that included hATTR and wtATTR cardiomyopathy patients, tafamidis was associated with lower all-cause mortality and rates of cardiovascular hospitalizations [13]. However, there was no significant difference in the baseline to 30 months variation of LV wall thickness or LV-EF between the tafamidis and placebo group as assessed by echocardiography [13].

Lastly, after the aforementioned tafamidis therapy but prior to LTx, the patient received inotersen within a double-blind randomized trial of inotersen vs. placebo. Inotersen is an antisense oligonucleotide inhibitor of the hepatic production of TTR that was shown to improve the course of neurologic disease and quality of life in patients with hATTR amyloidosis [14]. Since 2018, it has been approved for the treatment of polyneuropathy in these patients [3]. A small study of inotersen in patients with ATTR cardiomyopathy showed no relevant improvement in imaging parameters including LV wall thickness or mass on CMR, and echocardiography derived global systolic strain at 12 months. The respective authors hypothesized that inotersen might stabilize disease progression and improve life expectancy [15].

To conclude, we present the case of a hATTR patient manifesting with predominant neuropathy and presence of cardiomyopathy with regressive non-invasive imaging findings, as depicted by CMR within 5-year-follow-up time. It is difficult to differentiate to which extent this is due to one of the anti-amyloid therapies that were implemented in this case. Obviously, the mild cardiac clinical course as well as the noted cardiac phenotype “regression” are unlikely to be only a reflection of the natural history of the disease. Hence, we believe that either one of the aforementioned therapeutic approaches—or their combination—resulted in the depicted regression of cardiac involvement in this case. To the best of our knowledge, this is the first publication describing such findings in a patient with hATTR cardiomyopathy; until now a regression of imaging findings was reported only in the completely different setting of light-chain (AL) amyloid cardiomyopathy after stem cell transplantation [16].

## Funding sources

None.

## References

[1] Adams D, Koike H, Slama M, Coelho T (2019). Hereditary transthyretin amyloidosis: a model of medical progress for a fatal disease. Nat Rev Neurol.

[2] Emdin M, Aimo A, Rapezzi C et al. Treatment of cardiac transthyretin amyloidosis: an update. *Eur Heart J* 2019 May 20. 10.1093/eurheartj/ehz298 31111153

[3] Ruberg FL, Grogan M, Hanna M, Kelly JW, Maurer MS (2019). Transthyretin amyloid cardiomyopathy: JACC State-of-the-Art Review. J Am Coll Cardiol.

[4] Lane T, Fontana M, Martinez-Naharro A (2019). natural history, quality of life, and outcome in cardiac transthyretin amyloidosis. Circulation.

[5] Dahlem K, Michels G, Kobe C, Bunck AC, Ten FH, Pfister R (2017). Diagnosis of cardiac transthyretin amyloidosis based on multimodality imaging. Clin Res Cardiol.

[6] Damy T, Kristen AV, Suhr OB et al. Transthyretin cardiac amyloidosis in continental Western Europe: an insight through the Transthyretin Amyloidosis Outcomes Survey (THAOS). *Eur Heart J* 2019 April 1. 10.1093/eurheartj/ehz173 PMC8825236 30938420

[7] Gonzalez-Duarte A, Cardenas-Soto K, Banuelos CE (2018). Amyloidosis due to TTR mutations in Mexico with 4 distinct genotypes in the index cases. Orphanet J Rare Dis.

[8] Magy N, Valleix S, Grateau G (2002). Transthyretin mutation (TTRGly47Ala) associated with familial amyloid polyneuropathy in a French family. Amyloid.

[9] Algalarrondo V, Antonini T, Theaudin M (2018). Cause of death analysis and temporal trends in survival after liver transplantation for transthyretin familial amyloid polyneuropathy. Amyloid.

[10] Ericzon BG, Wilczek HE, Larsson M (2015). Liver Transplantation for Hereditary Transthyretin Amyloidosis: After 20 Years Still the Best Therapeutic Alternative?. Transplantation.

[11] Okamoto S, Zhao Y, Lindqvist P (2011). Development of cardiomyopathy after liver transplantation in Swedish hereditary transthyretin amyloidosis (ATTR) patients. Amyloid.

[12] Suhr OB, Larsson M, Ericzon BG, Wilczek HE (2016). Survival After Transplantation in patients with mutations other than Val30Met: extracts From the FAP World Transplant Registry. Transplantation.

[13] Maurer MS, Schwartz JH, Gundapaneni B (2018). Tafamidis Treatment for patients with transthyretin amyloid cardiomyopathy. N Engl J Med.

[14] Benson MD, Waddington-Cruz M, Berk JL (2018). Inotersen Treatment for patients with hereditary transthyretin amyloidosis. N Engl J Med.

[15] Benson MD, Dasgupta NR, Rissing SM, Smith J, Feigenbaum H (2017). Safety and efficacy of a TTR specific antisense oligonucleotide in patients with transthyretin amyloid cardiomyopathy. Amyloid.

[16] Brahmanandam V, McGraw S, Mirza O, Desai AA, Farzaneh-Far A (2014). Regression of cardiac amyloidosis after stem cell transplantation assessed by cardiovascular magnetic resonance imaging. Circulation.

